# Perceived confidence to use female condoms among students in Tertiary Institutions of a Metropolitan City, Southwestern, Nigeria

**DOI:** 10.1186/s13104-017-2730-6

**Published:** 2017-08-11

**Authors:** Taiwo A. Obembe, Ayo S. Adebowale, Kehinde O. Odebunmi

**Affiliations:** 1 Department of Health Policy & Management, Faculty of Public Health, College of Medicine, University of Ibadan, Ibadan, Nigeria; 2 Faculty of Health Sciences, University of Witwatersrand, Johannesburg, South Africa; 3 Department of Epidemiology and Medical Statistics, Faculty of Public Health, College of Medicine, University of Ibadan, Ibadan, Nigeria; 4 Department of Hospice and Palliative Medicine, University College Hospital, Ibadan, Nigeria

**Keywords:** Female condom, Barrier methods, Perceived confidence, Tertiary students, Undergraduates

## Abstract

**Background:**

Latex condoms for men have been documented to offer high efficacy as both a contraceptive and protection against sexually transmitted diseases. This equally establishes the importance of continued research on female condoms. This study aims to investigate the perceived confidence to use the female condoms amongst undergraduate female students from selected tertiary institutions from Ibadan Southwestern Nigeria.

**Methods:**

The study was a descriptive cross-sectional survey involving 388 female undergraduate students selected through a multistage sampling technique. The survey was carried using pre-tested semi-structured questionnaires. Quantitative data were analyzed using the Statistical Package for the Social Sciences to generate frequencies, cross tabulations of variables at 5% level of significance.

**Results:**

Mean age of respondents 18.26 ± 3.45 with most students being 20–24 years (55.2%), single (92.8%), Yorubas (85.6%) and from the polytechnic institutions (41.0%). Only 10.8% had good perceived confidence to use a female condom. Perceived confidence was significantly higher amongst other ethnicities (19.59 ± 3.827) compared to Yoruba ethnicity (18.04 ± 3.337) (F = 9.935; p < 0.05). Likewise, students from the Polytechnic campuses exhibited significantly higher mean scores (18.81 ± 3.187) compared to others (F = 3.724; p < 0.05). Perception towards the condom was a significant factor that influenced the confidence to use a female condom (F = 9.896; p < 0.000).

**Conclusions:**

Concerted efforts are advocated to improve the low perception exhibited towards the use of female condoms and the low perceived confidence to its utilization. This would help to transfer the decision making and control to women thus contributing to their empowerment and increased protection from unplanned pregnancies and sexually transmitted diseases.

## Background

The female condom first proposed by Lasse Hessel in mid-80s [1], is certified to guard against unwanted pregnancies and sexually transmitted diseases during vaginal intercourse [2, 3]. It’s distribution and availability worldwide has doubled from 25 million to 50 million and 60 million units in 2007, 2010 and 2012 respectively [4, 5].

Due to young women’s increased susceptibility to HIV and other sexually transmitted infections particularly prevailing gender norms that endorse male dominance in decision making with respect to sexual issues [6], it would be easily assumed that availability, uptake, and utilization of female condoms would be optimal. HIV-related infections account for most deaths among women between 15 and 49 years with close to 60% of the total global population of people living with HIV-infected women in sub-Saharan Africa [7] with gender inequality as a driving force of the epidemic [8]. In Nigeria, the prevalence of sexually transmitted diseases among Nigeria female youths is as high as 17% [9]. About 2.9 million people live with HIV/AIDs with a concurrent startling low rate of male condom use estimated at 25 and 11% for sexually active males and females respectively [10, 11]. Furthermore, about 8 out of every 10 infected women with HIV by having unprotected sex with an infected male partner. This is particularly important as women’s biological vulnerability to HIV through sexual intercourse is up to four times as high compared to the men. Yet, the subordinate disposition of women often offers very little control over whether a man uses a condom or not [1, 3].

The general uptake and utilization of the female condom remain inadequate with both men and women having mixed reactions about its use. For example, Malawian women hold a negative attitude towards its use [12]. Surveyed women attributed their main discouragement of use to fears of negative consequence, poor knowledge, non-user friendly and reduction in sexual pleasure [12–14]. In another study conducted in South Africa, main facilitators for usage and acceptance of female condom by men included the convenience of use, curiosity and enhanced sexual sensation while barriers ranged from insertion difficulties, concerns over loss of control and limited familiarity largely ranging from socio-economic to environmental factors [6, 15]. Despite the extensive literature on barriers and facilitators of condom use amongst both males and females, studies have not taken into cognizance the understanding of individual differences with regards to adoption and utilization of female condom as a health behavior [16]. The theory of reasoned action (with emphasis on self-efficacy) “relies on individual-focused cognitive models of rational decision making, which are explicitly abstracted from social context, to explain health-related behavior” [17]. Self-efficacy that refers to an individual’s perception of his or her competence to successfully perform a behavior [18], is a cogent factor requiring in-depth investigation when examining the sub-optimal uptake of promising health interventions such as the utilization of female condoms amongst women. Our study thus sought to examine the factors that influence the perceived confidence to use the female condom amongst the female undergraduate students from selected tertiary institutions in Southwestern Nigeria.

## Methods

The study employed a cross-sectional study design to measure perceived confidence to use a female condom during sexual encounters and its associated factors among undergraduate female students from selected tertiary institutions in Oyo State.

The study was conducted across selected tertiary institutions across Ibadan metropolis of Oyo State, Nigeria. Data were collected using a 57-item interviewer-administered questionnaire that consisted of demographic characteristics, sexual activity, transactional sex, awareness of female condom, perception (attitude) towards the female condom, perceived confidence (self-efficacy) to use the female condom and utilization of female condoms. Validity was first ensured by developing the questionnaire after a review of the literature prepared in English. It was then translated into Yoruba and back-translated to verify that the original meaning was not lost. We utilised four types of questions; binary questions (such as yes/no); multiple choice questions (in which the options were mutually exclusive and covered all possible answers); specific questions that do not specify options (for example how old are you?); and scaling questions (Likert scale was used to assess students perceived level of self-efficacy) [19]. The Flesch reading ease score 64.3 and the Coleman-Liau index of 8 (indicating that it was fairly easy to read) were used to ensure proper comprehension of our instrument [20, 21]. A pre-test of the questionnaire was carried out in 10% of the sample size in similar tertiary institutions (a university, polytechnic and a college of education) from a neighboring state, Osun State among 40 students before the actual data collection. Reliability testing of the scales yielded an overall Cronbach’s Alpha of 0.847 (95% CI 0.824–0.868). The self-efficacy scale that was used was adapted from technical, assertiveness, and sexual control components of ‘condom use self-efficacy scale’ proposed by Baele et al. [22] and condom use self-efficacy (CUSES-E) [23].

A minimum sample size of 259 was obtained using a 5% level of significance, at a 90% power and a 5% tolerable error. The prevalence of positive attitudes towards the use of a female condom (46%) amongst female undergraduate students was used as our reference value [24]. This was increased to 323 to adjust for a 20% non-response.

A multistage sampling technique was employed for recruitment of study participants. First, tertiary institutions in the state were divided into three strata: universities, polytechnics, and colleges of education. One institution was randomly selected from each stratum (total 3). In each selected institution, two female halls of residence were randomly selected (total 6). A full list of registered students from all selected halls of residence was obtained followed by a systematic random selection of 65 students per hall of residence. The rooms were systematically selected using the equiprobability method [25]. The 1st room was picked randomly between the rooms 1–3 of each hall after which the sampling interval was estimated by dividing the total number of rooms in the hall (population size) by 65 (sample size). This sampling interval was thereafter used to determine the rooms to be skipped or picked. In every room that was picked, only 1 student was randomly selected per room to be interviewed. This was done by asking all students to pick numbers randomly from a bag intermingled with a blank sheet; the student that picked a blank sheet was selected to participate in each room. All visitors, unlawful residents, and students that were not registered in the halls of residence were considered ineligible to be part of our study. This was verified by sighting both the identification cards issued by the institution and also by the hall of residence. Other eligibility criteria considered were consent of respondents to participate in the study and availability as at the time of the data collection. A total of 400 students were surveyed, however 12 entries were dropped at analysis due to improperly filled survey instruments. The questionnaire was designed to be self-completed.

Our independent variables included the age, marital status, religion, ethnicity, type of institution, family setting, and perception towards the female condom while the dependent variable is the “perceived confidence to use a female condom”. Perception towards the use of female condom and perceived confidence to use the female condom were assessed by 7-item and 11-item constructs respectively. Strongly agree, agree, indifferent, disagree and strongly disagree were scored 1, 2, 3, 4, 5 accordingly. Maximum obtainable scores (by summing up all items) for perception towards the female condom and perceived confidence to use the female condom are 30 and 55 respectively. Scores of ≥23, 15–22, and ≤14 for perception towards the female condom were re-categorized as good, fair and poor perception while scores of ≥42, 28–41, and ≤28 for perceived confidence were categorized as good, fair and poor. Associations between the dependent variable (mean scores of perceived confidence to use a female condom) and the independent variables were investigated using F test at 5% level of significance. Associations between the dependent variable (categorized perceived confidence to use a female condom) and the independent variables were investigated using Chi square test at 5% level of significance. Frequency tables were generated for relevant variables while descriptive statistics were used to summarize continuous variables (such as means and standard deviations).

Approval for the conduct of the study was obtained from the Ethics Review Committee of Oyo state Ministry of Health. Written informed consent was also obtained from the students’ prior to questionnaire administration and respondents’ anonymity was protected by ensuring that no individual identifiers existed in the instruments or in the electronic data set. Parental consent was also sought and obtained for participants younger than 16 years.

## Results

The ages of the respondents ranged from 14 to 36 years with a mean of 18.26 ± 3.45 years. More than half (55.2%) of our respondents were aged 20–24 years. Expectedly, the majority of our respondents were never married (92.8%), Christians (72.9%), from monogamous family settings (72.2%) and of Yoruba descent (85.6%) (Table 1). Students from Polytechnic institutions (41.0%) accounted for a greater proportion of our study participants followed closely by those from the universities (35.6%). Most students were in the 2nd year (36.9%) trailed closely by those in the 3rd year (25.5%). Students in the first year and 4 years and above were 20.6 and 17.0% respectively (Table 1).

**Table 1:** Sociodemographic characteristics of respondents (n = 388)

S/no.	Variable	Frequency (n)	Percentage (%)
1.	Age		
	14–19	104	26.8
	20–24	214	55.2
	25+	70	18.0
2.	Marital status		
	Never married	360	92.8
	Ever married/cohabiting	28	7.2
3.	Religion		
	Christianity	283	72.9
	Islam	105	27.1
4.	Ethnicity		
	Yoruba	332	85.6
	Others	56	14.4
5.	Type of institution		
	Polytechnic student	159	41.0
	University student	138	35.6
	College of education student	91	23.5
6.	Level of education		
	1st year	80	20.6
	2nd year	143	36.9
	3rd year	99	25.5
	4th year and above	66	17.0
7.	Family setting		
	Monogamous	280	72.2
	Polygamous	108	27.8

**Fig. 1:**
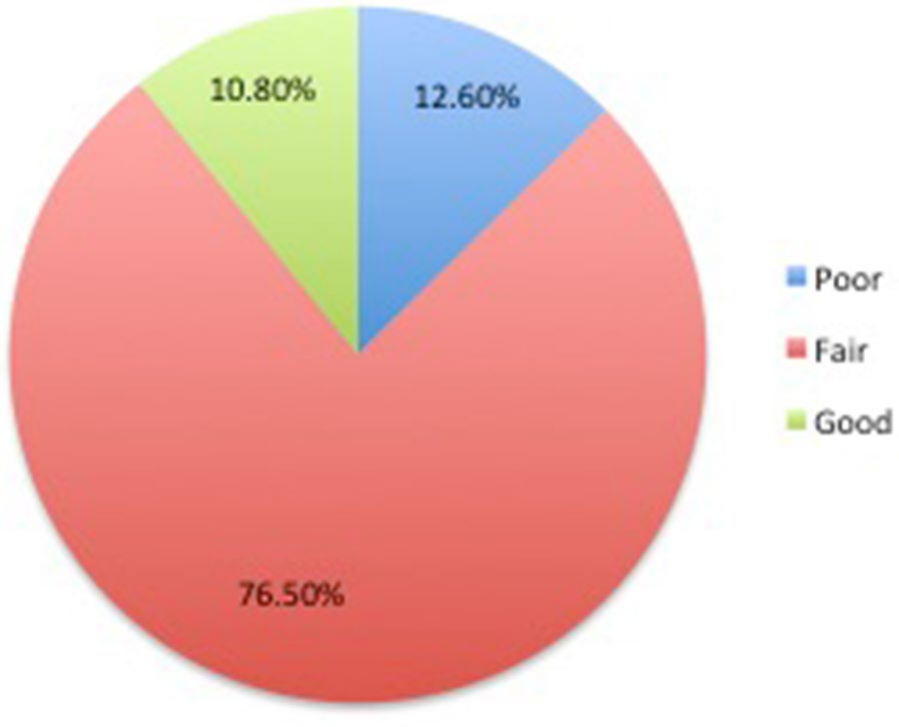
Perceived confidence to use the female condom

Most respondents demonstrated fair perception (76.5%) towards using the female condom while those that had good and poor perception were 10.8 and 12.6% respectively (Fig. 1).

Less than half of our participants (44.6%) felt bold/confident enough to use a female condom before a sexual intercourse. Amongst these, only 14.7% of these felt strongly of their abilities to use a female condom if the situation arose. About one-third (35.1%) attested that persuading their partners to accept the utilization of a female condom will be difficult based on health (STI/HIV) reasons while 43.6% participants disagree with the perception. Likewise, 39.7% of participants wouldn’t feel comfortable using female condom based on suspicions of infidelity while 41.7% disagreed with the idea. Some respondents also agreed that they would not be able to convince their partners to support the use of the female condom due to anger issues (38.1%). Others (42.5%) disagreed that temper and anger issues would not deter them from suggesting it to their partners. Only 31.4% could stop to insert a female condom during a sexual intercourse. Less than half of respondents (45.1%) believed that they can suggest and convince an unwilling partner to agreeing to accept the idea of a female condom (Table 2).

**Table 2:** Reasons influencing use of female condom (FC)

	N (%)	N (%)	N (%)	N (%)	N (%)
	Strongly agree	Agree	Indifferent	Disagree	Strongly disagree
1. If given the female condom, I will be bold/confidence enough to use it before intercourse	57 (14.7%)	116 (29.9%)	77 (19.8%)	92 (23.7%)	46 (11.9%)
2. I don’t think I can persuade my partner to accept the use of the female condom because he may feel I think he has an STI/HIV	53 (13.7%)	83 (21.4%)	83 (21.4%)	105 (27.1%)	64 (16.5%)
3. I wouldn’t feel comfortable using female condom because my partner may think It’s because I suspect him of infidelity	63 (16.2%)	91 (23.5%)	72 (18.6%)	118 (30.4%)	44 (11.3%)
4. I wouldn’t feel comfortable using female condom because of fear that my partner may get furious or lose his temper	42 (10.8%)	106 (27.3%)	75 (19.3%)	109 (28.1%)	56 (14.4%)
5. I can stop to insert a female condom during the act	51 (13.1%)	71 (18.3%)	94 (24.2%)	116 (29.9%)	56 (14.4%)
6. I can suggest and initiate use of female condom to an unwilling partner	67 (17.3%)	108 (27.8%)	80 (20.6%)	70 (18.0%)	63 (16.2%)

Perceived confidence as exhibited by teenagers 14–19 years (18.37 ± 3.484) and young adults 20-24 years (18.37 ± 3.430) were higher than those above 25 years (17.76 ± 3.466) of age though not significant (F = 0.908; p = 0.404). Participants that were never married (18.30 ± 3.422) also were perceived to be more confident compared to those that were “ever married” (17.75 ± 3.826) also not a significant finding (F = 0.660; p = 0.417) (Table 3). Mean scores for perceived confidence amongst Christians (18.34 ± 3.484) was marginally higher than the combined scores from Muslims (18.04 ± 3.362) (F = 0.597; p = 0.440). Perceived confidence was significantly higher amongst other ethnicities (19.59 ± 3.827) compared to Yoruba ethnicity (18.04 ± 3.337) (F = 9.935; p = 0.02). Likewise, significant differences in mean scores were observed amongst the students based on the type of institution in which they were schooling. Students from the Polytechnic campuses exhibited higher scores (18.81 ± 3.187) compared to University students (18.03 ± 3.683) and students from college of education (17.66 ± 3.426) (F = 3.724; p = 0.025) (Table 3). The mean scores for perceived confidence amongst students from the second year (18.57 ± 3.528) were higher than those of 1st year (18.50 ± 3.265), 4th-year students (18.15 ± 3.592) and those from the 3rd year (17.70 ± 3.364) (F = 1.410; p = 0.239). Type of family setting from which the participants hailed from also showed an impact on perceived confidence though not significant. Those from monogamous family settings were seen to possess higher confidence scores (18.34 ± 3.454) compared to their counterparts from polygamous family settings (18.06 ± 3.449) (F = 0.480; p = 0.489). Perception towards the female condom had a very significant association with how students perceived their confidence to use the female condom when the situation demanded it. Mean scores for students with good perception (18.75 ± 3.237) were significantly higher than those with fair perception (18.66 ± 3.519) and poor perception (16.87 ± 3.061) respectively (F = 9.896; p < 0.000) (Table 3).

**Table 3:** Association between sociodemographic variables and mean scores for perceived confidence to use the female condom

		Perceived confidence to use a female condom			
		Mean (SD)	Mean square	F	p
1.	Age				
	14–19	18.37 ± 3.484	10.814	0.908	0.404
	20–24	18.37 ± 3.430			
	25+	17.76 ± 3.466			
2.	Marital status				
	Never married	18.30 ± 3.422	7.859	0.660	0.417
	Ever married	17.75 ± 3.826			
3.	Religion				
	Christianity	18.34 ± 3.484	7.108	0.597	0.440
	Islam	18.04 ± 3.362			
4.	Ethnicity				
	Yoruba	18.04 ± 3.337	115.589	9.935	0.002*
	Others	19.59 ± 3.827			
5.	Type of institution				
	Polytechnic student	18.81 ± 3.187	43.715	3.724	0.025*
	University student	18.03 ± 3.683			
	College of education	17.66 ± 3.426			
6.	Level of education				
	First year	18.50 ± 3.265	16.732	1.410	0.239
	Second year	18.57 ± 3.528			
	Third year	17.70 ± 3.364			
	Fourth year	18.15 ± 3.592			
7.	Family setting				
	Monogamous	18.34 ± 3.454	5.720	0.480	0.489
	Polygamous	18.06 ± 3.449			
8.	Perception towards female condom				
	Poor	16.87 ± 3.061	112.623	9.896	0.000*
	Fair	18.66 ± 3.519			
	Good	18.75 ± 3.237			

* Significant associations

With respect to age groups with good perceived confidence, more young adults (20–24 years) had good perceived confidence to use the female condom (52.4%) compared to the teenagers 14–19 years (28.6%) and those 25+ years (19.0%) respectively (X^2^ = 4.588, p = 0.332) (Table 4). More participants that were never married had good perceived confidence (92.8%) when compared to those ever married/cohabiting (7.2%) (X^2^ = 4.250; p = 0.119). More Christians (73.8%) compared to Muslims (26.2%) had good perceived confidence in using the female condom (X^2^ = 0.361; p = 0.835). Significantly more females of Yoruba ethnicity (71.4%) had good perceived confidence to use a female condom compared to participants from other ethnicities (28.6%) (X^2^ = 7.915; p = 0.019) (Table 4). Similarly, more polytechnic students (45.2%) demonstrated good perceived confidence to use the female condom compared to the participants from the university (40.5%) and those from the colleges of education (14.3%); though not a significant association (X^2^ = 6.484; p = 0.166) (Table 4). More respondents in the 2nd year (45.2%) had good perceived confidence to use the female condom compared to students in other levels though not a significant finding (X^2^ = 6.667; p = 0.353). More students from monogamous family settings (76.2%) had good perceived confidence compared to those from polygamous settings (23.8%) (X^2^ = 1.533; p = 0.465) (Table 4). Significantly more respondents with fair perception (80.9%) towards the female condom showed good perceived confidence with respect to other cadres (X^2^ = 27.755; p < 0.000) (Table 4).

**Table 4:** Correlates between socio-demographic variables and perceived confidence to use the female condom

		Perceived confidence to use female condom				
		Poor	Fair	Good	X^2^	p
1.	Age (years)					
	14–19	12 (24.5%)	80 (26.9%)	12 (28.6%)	4.588	0.332
	20–24	23 (46.9%)	169 (56.9%)	22 (52.4%)		
	25+	14 (28.6%)	48 (16.2%)	8 (19.0%)		
2.	Marital status					
	Never married	42 (85.7%)	279 (93.9%)	39 (92.8%)	4.250	0.119
	Ever married/cohabiting	7 (14.3%)	18 (6.1%)	3 (7.2%)		
3.	Religion					
	Christianity	34 (69.4%)	218 (73.4%)	31 (73.8%)	0.361	0.835
	Islam	15 (30.6%)	79 (26.6%)	11 (26.2%)		
4.	Ethnicity					
	Yoruba	44 (89.8%)	258 (86.9%)	30 (71.4%)	7.915	0.019*
	Others	5 (10.2%)	39 (13.1%)	12 (28.6%)		
5.	Type of institution					
	Polytechnic student	14 (28.6%)	126 (42.4%)	19 (45.2%)	6.484	0.166
	University student	23 (46.9%)	98 (33.0%)	17 (40.5)		
	College of education student	12 (24.5%)	73 (24.6%)	6 (14.3%)		
6.	Level of education					
	First year	7 (14.3%)	64 (21.5%)	9 (21.4%)	6.667	0.353
	Second year	15 (30.6%)	109 (36.7%)	19 (45.2%)		
	Third year	17 (34.7%)	76 (25.6%)	6 (14.3%)		
	Fourth year and above	10 (20.4%)	48 (16.2%)	8 (19.1%)		
7.	Family setting					
	Monogamous	32 (65.3%)	216 (72.7%)	32 (76.2%)	1.533	0.465
	Polygamous	17 (34.7%)	81 (27.3%)	10 (23.8%)		
8.	Perception towards female condom					
	Poor	24 (49.0%)	62 (20.9%)	3 (7.1%)	27.755	0.000*
	Fair	19 (38.8%)	182 (61.3%)	34 (80.9%)		
	Good	6 (12.2%)	53 (17.8%)	5 (12.0%)		

* Significant associations

## Discussion

It is not surprising that majority of our participants are between 14 and 24 years with a mean age that corresponds closely with school age in tertiary institutions in Nigeria [26]. The majority of our participants being single also corroborates the national estimates that put median age at first marriage for females at 18.1 years [27]. Similarly, findings such as the preponderance of respondents being Yorubas and Christians also can be explained logically by the fact that the study was carried out in the capital city of Oyo State inhabited predominantly by such [28].

The desire for lifetime partners or “*true love*” amongst older participants may well explain the lower perceived confidence to negotiate with partners on sexual matters [29]. Lack of trust, loss of control, the likelihood of domestic violence, infidelity were highlighted as issues raised by our respondents that would reduce their perceived confidence in the event of unplanned sexual encounters ratifies what is documented in other studies [14, 30, 31].

Only 35.1% of our respondents can negotiate with sexual partners on the need and importance of a female condom for sexual activities which is in contrast to findings of another study where about 73.0% of female undergraduates had been engaged in sexual negotiations before [32]. Adebiyi and Asuzu argue that such circumstances that hinder condom use are mostly fostered within relationships where the opportunity for discussion on sexual matters is non-existent [13] and advocates for a need to conduct more studies that focus on communication within sexual partnerships. The increased perceived confidence by other ethnicities to use the female condom relatively to Yoruba contradicts the literature [33]. Though this contradiction may be because our study focused on women alone as opposed to male-only respondents that Ankomah and colleagues surveyed [33]. Also contrary to our findings, Ayoola et al. found southeastern women to possess a reduced capacity to negotiate condom use with their partners [34]. Studies that probe for reasons why other ethnicities have higher perceived confidence to Yorubas are highly desirable considering the relative literacy of the Yorubas compared to other ethnicities [27]. Likewise, the exhibition of significantly higher mean scores amongst polytechnic students compared to other institutions calls for deeper studies. Lax family ties that are commoner with polygamous family settings [35, 36] can explain why mean scores for perceived confidence of respondents from monogamous family settings were higher than those from polygamous family settings.

Even though our study primarily surveyed individual level factors as opposed to socio-economic and environmental factors influencing condom use, poor perception towards female condom use still supports findings from literature in which perceptions that influenced the use of condoms were varied [15]. This finding is also mirrored in health settings where providers in South Africa were less likely to counsel women on female condoms but were more likely to counsel women on male condoms [37]. The low perceived confidence to use the female condom may translate well to the poor utilization of the female condom which is reiterated in other studies [14, 27]. Apart from logistics which considerably impedes the rate of availability of female condoms [37, 38], other factors may well be responsible for this poor utilization such as difficulties with insertion [39].

As with cross-sectional surveys, our study can elucidate factors associated with the use of the female condom, but it cannot establish causation. Our study failed to examine the relationship between some important variables such as socio-economic status on perceived confidence to use the female condom among the undergraduate students. It will be desirable to explore such relationships in further studies. Our study could have yielded more robust findings if a supportive qualitative study had been carried out to probe the reasons for the different levels of the perceived confidence to use the female condom across the different tertiary institutions. Qualitative studies and triangulation of findings should be undertaken in subsequent studies in order to establish a deeper understanding of the factors influencing such variations in outcome of interest. The generalizability of our results is limited due to the factors chosen for our analysis. In addition, this study did not consider service related issues [40] that contribute to the perceived confidence in its usage.

## Policy issues

Considering the grossly unmet need for contraception, coupled with the absence of female-led and female-initiated alternatives to the female condom, stronger policies are required to explore the advantages of female condom towards reducing the scourge of HIV/AIDs and sexually transmitted diseases in sub-Saharan Africa [41]. Female condoms, not only help to transfer control to women in sexual encounters but also assists the 5th goal in the sustainable development goals which aims to empower women [42].

Studies have reiterated that the concurrent availability of female condoms results in higher rates of protected sex, greater negotiating power and control of their health compared with only-male condom availability and the affordability [5]. This availability will be greatly enhanced if policies that promote subsidization of costs (for developing countries in sub-Saharan Africa where there are high prevalent rates of HIV infection) or that advocate free female condoms are embraced [3, 43].

The promise of an increased acceptance and uptake of the female condom when basic barriers are removed is certain, particularly when free trial samples are integrated into standard clinical practice and public health programs [44]. Ensuring dual availability of both types of condoms not only offers more protection but also improves the cost-effectiveness for preventing new HIV and sexually transmitted infections.

However, despite all these initiatives, the Federal Government of Nigeria has not taken any major leap towards increasing the availability of female condoms or its subsidization. Even though policy makers realize the need for revision and re-positioning of the National Health Policy to attain Sustainable Development Goals [45, 46], there is very little documentation or plan for scale up of availability of the female condom in the Nigerian Country Cooperation Strategic policy document that was eventually developed [47].

At the global level, more advocacy is also urgently required to reorient AIDS policy actors from viewing the AIDS scourge in the context of gender and reproductive health instead of sexuality and sexual rights [8]. Lastly, female-initiated barrier alternatives such as vaginal microbes and diaphragms that are non-toxic and sexual health services that are integrated into maternal health care and reproductive services are added initiatives [48] that can be explored at the global and national forums.

## Conclusions

The female condom offers better promises in the fight against HIV/AIDs and STD in Nigeria and greater sub-Saharan Africa if the availability of the female condom is buffered up and awareness campaigns to promote the populace on the benefits are scaled up. Sustained political will to subsidize costs of female condoms is equally needed to protect against risky sexual behaviors that are not uncommon occurrences with undergraduates of tertiary institutions.

## Abbreviations

FC: female condom
HMO: Health Maintenance Organization
HIV: human immunodeficiency syndrome
STD: sexually transmitted diseases
STI: sexually transmitted infections
